# Boerhaave’s syndrome: food particles in chest tube

**DOI:** 10.11604/pamj.2017.27.100.12890

**Published:** 2017-06-08

**Authors:** Lam Nguyen Ho, Hang Le Van

**Affiliations:** 1 Department of Internal Medicine, University of Medicine and Pharmacy-Ho Chi Minh city, Vietnam

**Keywords:** Boerhaave´s syndrome, methylene blue, tension pneumothorax

## Image in medicine

A 61-year-old alcoholic man presented to the emergency department because of vomiting and dyspnea after discharged from hospital due to appendectomy two days ago. He smoked heavily and his past medical history revealed gastritis. On the examination, he was hypotension (50/00 mmHg) and lethargy. Cardiopulmonary resuscitation was initiated aggressively. His chest x-ray showed right-sided tension pneumothorax (A). Chest drainage tube was inserted immediately and his clinical status improved spectacularly. On the fifth hospital day, the presence of food particles (rice, green onions) (red arrows) in the chest tube was found accidentally (B). Suspecting for esophageal perforation, the oral methylene blue test showed that the fluid in the chest tube turned bluish (C). Boerhaave's syndrome was established. An operation undertaken revealed a perforation of lower esophagus which was marked with nasogastric tube (yellow arrow) (D). However, his condition became worsen with nosocomial infection and died after that. The contents in the chest tube which can be an important diagnostic clue should be checked closely in the clinical practice.

**Figure 1:**
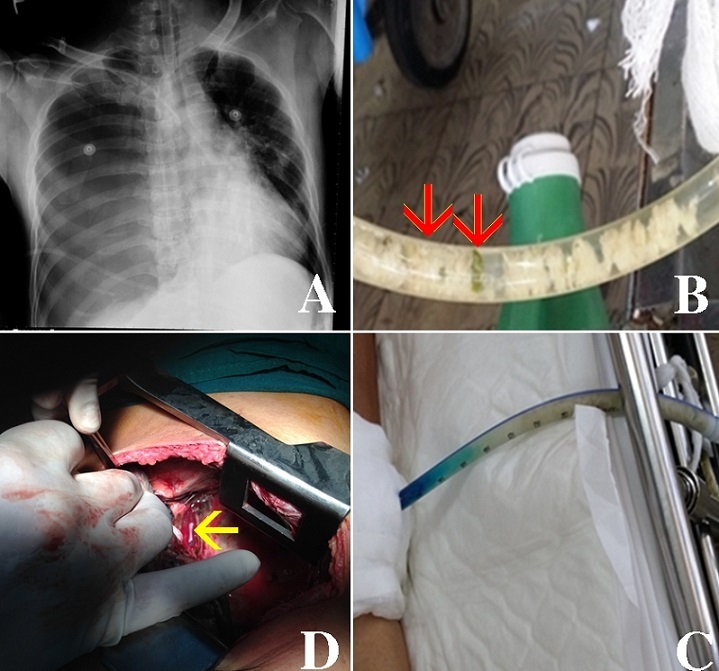
Boerhaave’s syndrome with food particles in chest tube: (A) chest x-ray showed right-sided tension pneumothorax; (B) rice and green onions (red arrows) appeared in the chest tube; (C) methylene blue which was swallowed came out through the chest tube; (D) a part of the nasogastric tube (yellow arrow) was found during the thoracotomy process

